# Supplementary material for: Anthropogenic climate change has altered primary productivity in Lake Superior
Source: Nat Commun. 2017 Jun 9;8:15713. doi: 10.1038/ncomms15713 (PMC5472714; doi:10.1038/ncomms15713)
Supplement: Supplementary Information — Supplementary Tables, Supplementary Discussion, Supplementary Figures, and Supplementary References [file ncomms15713-s1.pdf]

**Supplementary Tables**

**Supplementary Table 1**. Core location data listed for the three piston sediment cores taken from the Lake Superior basin.

| CORE | Year Taken | Water Depth (m) | Core Location | |
|---|---|---|---|---|
| | | | Latitude - N | Longitude - W |
| **Split Rock – SR** | 2002 | 250 | 47° 8' 25.8" | 91° 21' 3" |
| **Keweenaw – KW** | 2009 | 127 | 47° 7' 42" | 87° 49' 15.6" |
| **Isle Royale – IR** | 2011 | 233 | 47° 58' 24.8" | 88° 28' 6.24" |

**Supplementary Table 2**. Data listed for the eight sediment multicores. Mass accumulation rates (MARs) have not been constant across Lake Superior over the last 200 years. The far-right columns display the varying years encompassed by the bioturbation depth in each core and the %TOC offset used to fit the modeled diagenetic profiles of TOC for each multicore.

| CORE | Year Taken | Water Depth (m) | Core Location | | MARs | | Bioturbation Zone | | Offset |
|---|---|---|---|---|---|---|---|---|---|
| | | | Latitude N | Longitude W | $g/cm^2/yr$ | Depth (cm) | Depth (cm) | Years | %TOC |
| **BH03-3** | 2003 | 247 | 47° 53' 57" | 89° 39' 35" | 0.0175 | 0-10 | 1.25 | 9 | +0.6 |
| **BH09-2** | 2009 | 127 | 47° 7' 4" | 87° 49' 12" | 0.0252 | 0-20 | 1.5 | 9 | 0 |
| **BH09-3** | 2009 | 222 | 47° 35' 24" | 87° 50' 48" | 0.0135<br>0.0193 | 0-4<br>4-20 | 1.5 | 17 | 0 |
| **BH09-4** | 2009 | 256 | 47° 24' 42" | 90° 57' 24" | 0.0179<br>0.0269 | 0-5<br>5-20 | 1.5 | 9 | +1 |
| **LG MC** | 2010 | 213 | 47° 12' 39" | 85° 7' 39" | 0.0336<br>0.0269 | 0-18<br>18-25 | 2.0 | 4 | -0.3 |
| **IR MC** | 2010 | 234 | 47° 58' 23" | 88° 27' 58" | 0.0161<br>0.0359<br>0.0135 | 0-4<br>4-8<br>8-15 | 1.5 | 11 | +0.4 |
| **EM MC** | 2010 | 218 | 47° 33' 43" | 86° 35' 24" | 0.0091 | 0-8 | 1.0 | 13 | 0 |
| **CM MC** | 2010 | 252 | 48° 4' 35" | 87° 46' 26" | 0.0072 | 0-7 | 1.0 | 18 | -1.7 |

**Supplementary Table 3.** Paleomagnetic Secular Variation (PSV) inclination features[1,2] with corresponding depth, cal BP, and age associated error for the Split Rock piston core.

| **SPLIT ROCK** | | | |
|---|---|---|---|
| FEATURE | DEPTH (m) | AGE (cal BP) | Error of Age (±s.d.) |
| 5 | 0.56 | 2065 | 95 |
| 6 | 0.89 | 2610 | 150 |
| 7 | 1.09 | 3655 | 185 |
| 8 | 1.85 | 4300 | 350 |
| 9 | 2.33 | 4975 | 225 |
| 10 | 3.26 | 7225 | 195 |
| 11 | 3.74 | 7580 | 80 |
| 12 | 4.12 | 7760 | 80 |
| Glacial Varves | 4.97 | 9485 | 65 |
| Regression | | y = 1639.6x + 1361.8 | |
| Pearson's r | | 0.9850 | |

**Supplementary Table 4.** Paleomagnetic Secular Variation (PSV) inclination features[1,2] with corresponding depth, cal BP, and age associated error for the Keweenaw piston core.

| **KEWEENAW** | | | |
|---|---|---|---|
| FEATURE | DEPTH (m) | AGE (cal BP) | Error of Age (±s.d.) |
| 5 | 0.44 | 2065 | 95 |
| 6 | 0.58 | 2610 | 150 |
| 7 | 1.38 | 3655 | 185 |
| 8 | 1.69 | 4300 | 350 |
| 9 | 2.69 | 4975 | 225 |
| 10 | 4.09 | 7225 | 195 |
| 11 | 4.35 | 7580 | 80 |
| Mazama Ash | 4.4 | 7627 | 150 |
| 12 | 4.64 | 7760 | 80 |
| 13 | 4.91 | 8625 | 425 |
| Glacial Varves | 5.37 | 9485 | 65 |
| Regression | | y = 1386.9x + 1636.6 | |
| Pearson's r | | 0.9901 | |

**Supplementary Table 5.** Paleomagnetic Secular Variation (PSV) inclination features[1,2] with corresponding depth, cal BP, and age associated error for the Isle Royale piston core.

| **ISLE ROYALE** | | | |
|---|---|---|---|
| FEATURE | DEPTH (m) | AGE (cal BP) | Error of Age (±s.d.) |
| 2 | 0.43 | 1205 | 135 |
| 4 | 0.8 | 1610 | 100 |
| 5 | 1.32 | 2065 | 95 |

| | | | |
|---|---|---|---|
| 6 | 1.59 | 2610 | 150 |
| 9 | 1.88 | 4975 | 225 |
| 10 | 2.51 | 7225 | 195 |
| 11 | 2.65 | 7580 | 80 |
| Mazama Ash | 2.66 | 7627 | 150 |
| 12 | 2.79 | 7760 | 80 |
| 13 | 3.10 | 8625 | 425 |
| Glacial Varves | 3.44 | 9485 | 65 |
| Regression | $y = 177.56x^5 - 1850.6x^4 + 6500.7x^3 - 8429.3x^2 + 5227.3x + 30.217$ | | |
| Pearson's r | 0.9946 | | |

**Supplementary Discussion**

The modeled TOC profiles are similar (Figure 4 in main text) to those that we measured directly, which allows for the possibility that the profiles may be the result of steady-state degradation alone. The fits, however, were obtained with two adjustable parameters – $C(t_0)$ and $t_0$ – and are thus not unique. Additional degrees of freedom can be considered (for a better fit) if one accounts for the slight differences in the aerobic vs. anaerobic mineralization rates (as described in reference 3). Thus, the TOC data must be viewed with discretion and interpreted within the context of additional proxy data. When used in conjunction with other proxy data (i.e., $\delta^{13}C_{org}$ and $C_{org}$:N) the upcore increases in TOC lend somewhat token support to the proposed increase in autochthonous primary production driving the changes observed across the Lake Superior basin. As discussed in the main text, while significant changes in TOC concentrations can be expected during early diagenesis, the stable isotope composition of sedimentary organic carbon ($\delta^{13}C_{org}$) should remain unaltered – leaving a record of the original signal without the influence of diagenesis. Therefore, we use the $\delta^{13}C_{org}$ record as a more reliable indicator of changes in primary production (additionally discussed in the main text).

Shifts in phytoplankton community structure to greater abundances of $^{13}C$-rich organisms (e.g., diatoms[4]) can alter the isotopic signature (e.g., cause $^{13}C$-enrichment) of bulk organic matter deposited in sediments without actually increasing primary production. Diatom biovolume measurements in sediment cores LG MC and IR MC[6] reveal periods of enhanced diatom productivity but do not correspond to increases in $\delta^{13}C_{org}$. Indeed, application of a Pearson (two-tailed, $\alpha=0.05$) correlation shows that in LG MC biovolume and $\delta^{13}C_{org}$ records are not significantly correlated ($n=25$, $p>0.05$); whereas in IR MC the two records are negatively correlated ($r=-0.66$, $n=20$, $p<0.01$). Additionally, the ratio of centric to pennate diatom taxa in the two cores does not significantly vary through time (not shown). Benthic taxa are rare in these two cores[5], but another study[6] (limited to a single coring site in eastern Lake Superior) found that both planktonic and benthic taxa have increased simultaneously during the last ca. 50 years. These findings negate any potential influence from a switch between benthic and planktonic diatom taxa. Thus, while diatoms may be a contributing factor, it is not likely that the abundance of $^{13}C$-rich diatoms, changes in cell size/shape, or switch between benthic and planktonic taxa are the chief factors producing the lake-wide $^{13}C$-enrichment of bulk sedimentary organic matter observed between 1900 and present. Shifts in the historical diatom assemblages appear to be driven by phosphorus levels[6], whereas recent periods of lower diatom abundance appear to be balanced by relatively higher cyanophyte abundance[7].

Changes in diatom taxa in Lake Superior particularly since the 1980s are consistent with the influences of warming surface water temperatures including longer ice-free periods and prolonged thermal stratification documented in Lake Superior[5,8]. Additionally, decreases in water column silica concentrations[9] provide some evidence of increased primary production in Lake Superior between 1992 and 2010, possibly due to the same climate effects mentioned above. It

was ultimately concluded that the proposed increases in primary production were equivocal, as substantiation of any trends would require a longer time series than the ca. 20 years covered by the study[9]. Nevertheless, when all things are considered, the shifts in diatom taxa and decreases in water column silica concentrations support our conclusions (and vice versa) of increased primary production and influence of anthropogenic climate change on Lake Superior processes.

## Supplementary Figures

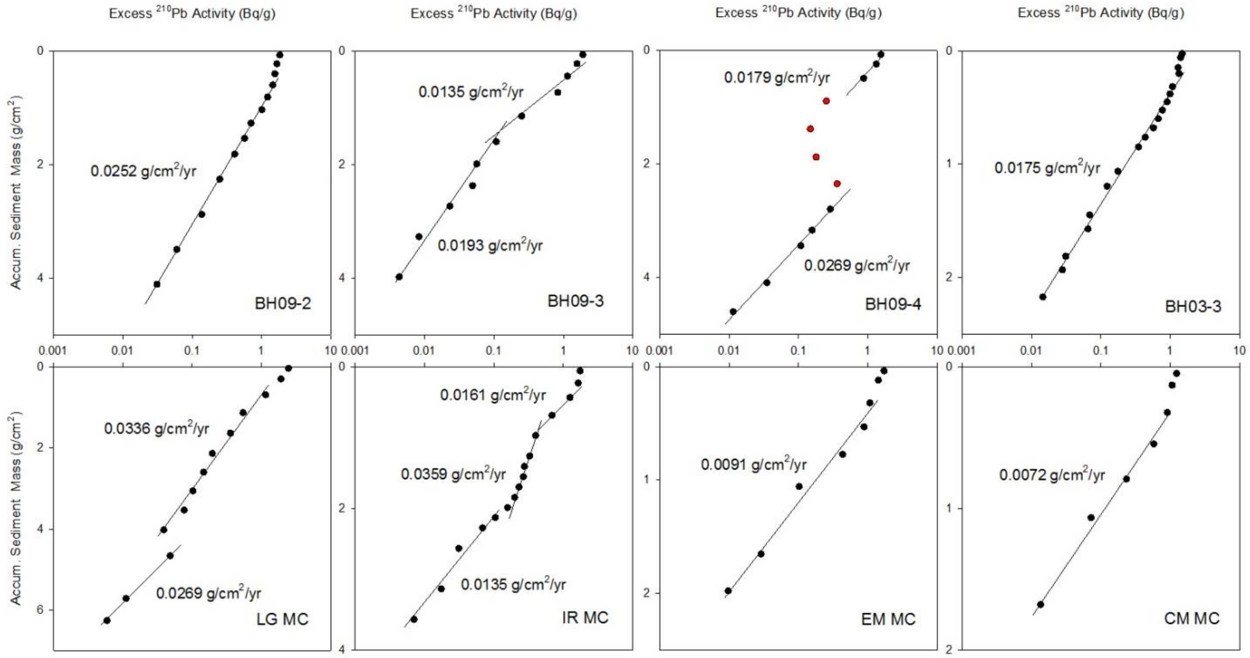


**Supplementary Figure 1**. Excess $^{210}$Pb vs. accumulated sediment mass in each core. The sediment mass accumulation rates (MARs) presented correspond to the linear segments that were fitted to the data illustrated in the linear portions of the eight cores from Lake Superior. The excursion seen in BH09-4, indicated in red, is attributed to the effects of ore processing off Silver Bay. *Note varying vertical scales.

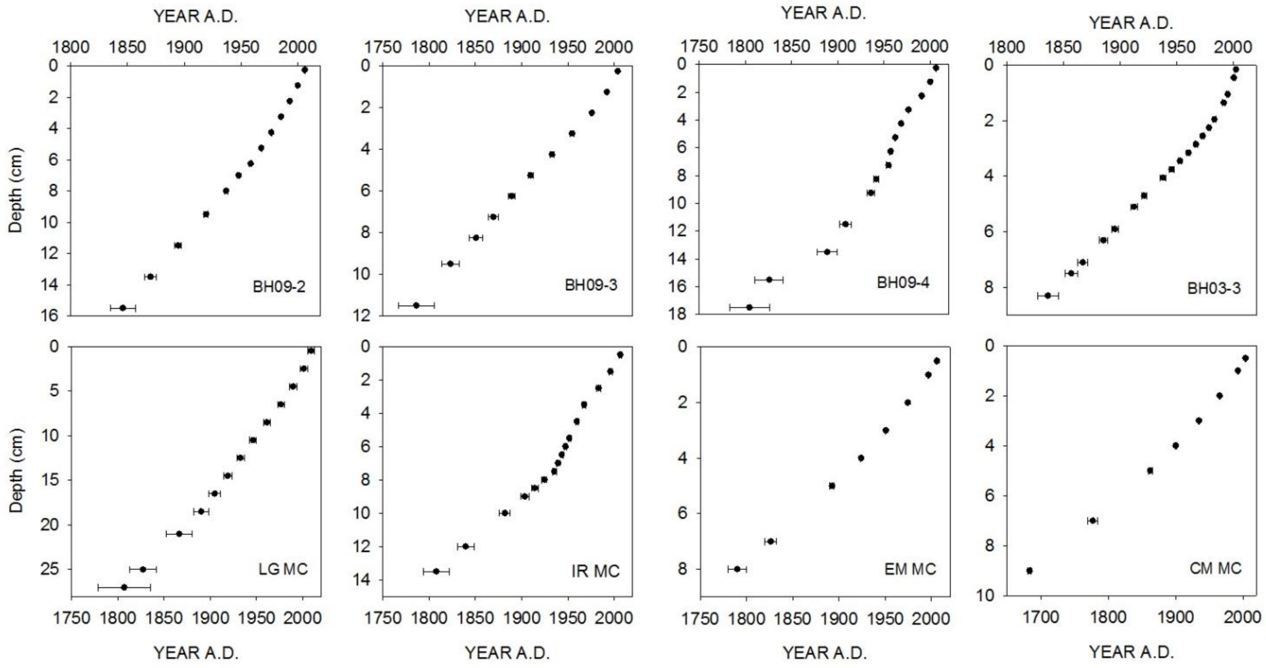


**Supplementary Figure 2.** Age vs. depth plots with age associated error. Where no error bars are observed, the symbol is larger than the associated error (± s.d.) at that depth. *Note varying vertical scales.

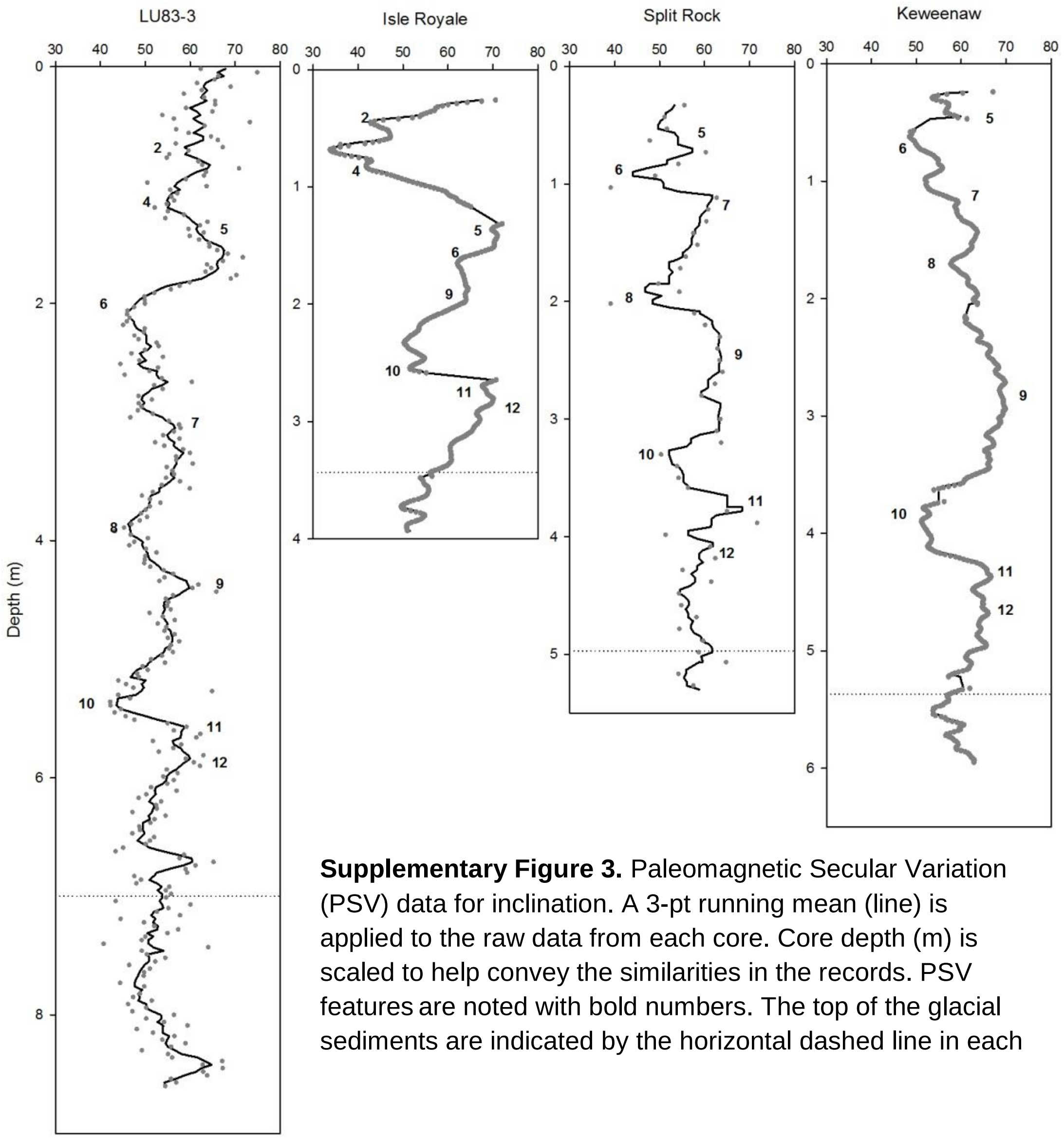


**Supplementary Figure 3.** Paleomagnetic Secular Variation (PSV) data for inclination. A 3-pt running mean (line) is applied to the raw data from each core. Core depth (m) is scaled to help convey the similarities in the records. PSV features are noted with bold numbers. The top of the glacial sediments are indicated by the horizontal dashed line in each

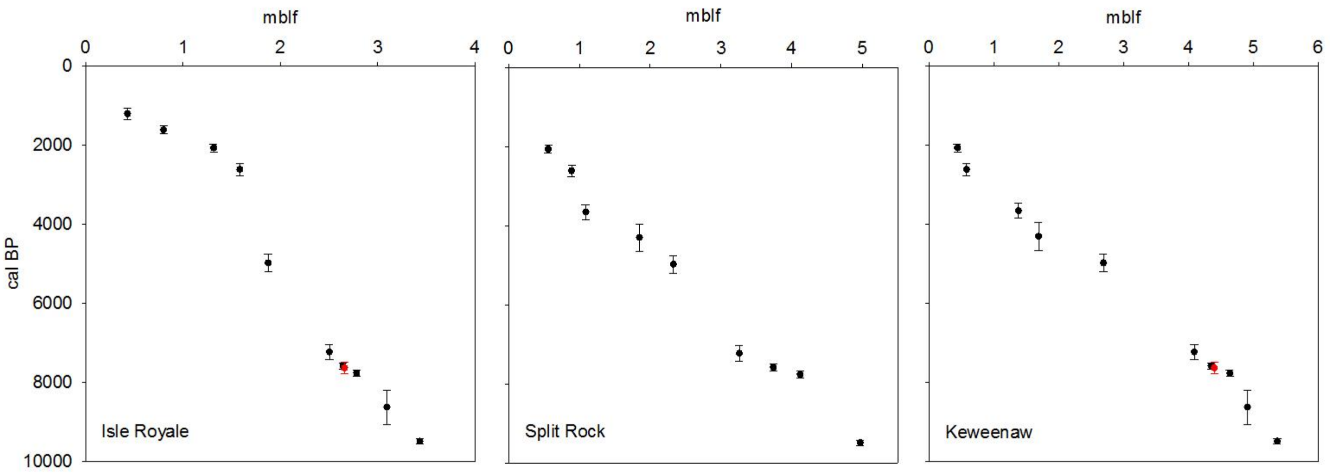


**Supplementary Figure 4.** Age-depth profiles with age associated error (± s.d.). Red symbol denotes age and depth of the Mazama Ash cryptotephra layer in cores Isle Royale and Keweenaw.

## Supplementary References


1. Breckenridge, A. & Johnson, T.C. Paleohydrology of the upper Laurentian Great Lakes from the late glacial to early Holocene. *Quat. Res*. **71,** 397-408 (2009).
2. Mothersill, J.S. Paleomagnetic dating of late glacial and postglacial sediments in Lake Superior. *Can. J. Earth Sci.* **25,** 1791–1799 (1988).
3. Katsev, S. & Crowe, S.A. Organic carbon burial efficiencies in sediments: The power law of mineralization revisited. *Geology* **43,** 607-610 (2015).
4. Fry, B. & Wainwright, S.C. Diatom sources of 13C-rich carbon in marine food webs. *Mar. Ecol. Prog. Ser.* **76,** 149-157 (1991).
5. Shaw Chraïbi, V.L., Kireta, A.R., Reavie, E.D., Cai, M. & Brown, T.N. A Paleolimnological Assessment of Human Impacts on Lake Superior. *J Great Lakes Res.* **40,** 886-897 (2014).
6. Stoermer, E.F., Kociolek, J.P., Schelske, C.L. & Conley, D.J. Siliceous microfossil succession in the recent history of Lake Superior. *Proc. Acad. Nat. Sci. Philad.* **13**7, 106-118 (1985).
7. Fahnenstiel, G. L., Sicko-Goad, L., Scavia, D. & Stoermer, E. F. Importance of picoplankton in Lake Superior. *Can. J. Fish. Aquat. Sci.* **43,** 235-240 (1986).
8. Reavie, E.D. *et al.* Climate warming and changes in *Cyclotella sensu lato* in the Laurentian Great Lakes. *Limnol. Oceanogr.* doi:10.002/lno.10459 (2016).
9. Barbiero, R.P., Lesht, B.M. & Warren, G.J. Convergence of trophic state and the lower food web in Lakes Huron, Michigan, and Superior. *J. Great Lakes Res.* **38,** 35-49 (2012).
